# Emerging Techniques and Treatment Outcomes in Pulmonary Arteriovenous Malformations Embolisation: A Narrative Review

**DOI:** 10.3390/jcm14238455

**Published:** 2025-11-28

**Authors:** Chai Jin Lim, Yousef Shahin

**Affiliations:** 1 Medical School, University of Sheffield, Sheffield S10 2RX, UK; cjlim1@sheffield.ac.uk; 2 Faculty of Health, University of Sheffield, Sheffield S10 2TN, UK; 3 Sheffield Vascular Institute, Sheffield Teaching Hospitals, Sheffield S10 2JF, UK

**Keywords:** pulmonary arteriovenous malformation (PAVM), embolisation, treatment outcomes, interventional radiology

## Abstract

**Background**: Pulmonary arteriovenous malformations (PAVMs) are abnormal vascular connections between the pulmonary arteries and veins, often leading to significant clinical complications. Embolisation has become the primary therapeutic modality for managing symptomatic PAVMs, with advancements in both technique and technology improving patient outcomes. Recent progress includes the introduction of precisely targeted embolisation techniques, such as cone-beam computed tomography (CBCT) guidance and three-dimensional imaging, enabling more accurate identification and treatment of complex, multiple, or peripheral lesions. Additionally, vascular plugs and microcoils have demonstrated superior performance in terms of lower recurrence rates and more complete occlusion of feeding vessels compared to traditional devices. The use of endovascular navigation systems has further enhanced procedural success. **Aim**: The objective of this review is to explore the latest innovations in embolisation therapies for PAVMs, emphasising emerging techniques, devices, and strategies that have refined treatment efficacy and safety. **Methods**: We performed a focused literature search to identify key publications relevant to current approaches in thoracic vascular imaging. Searches were conducted in PubMed and Embase using combinations of terms related to ‘thoracic imaging,’ ‘vascular,’ ‘Computed Tomography (CT) angiography,’ and ‘MRI.’ Additional articles were identified from reference lists of major reviews and landmark studies. Priority was given to publications from the past 10 years, with older key papers included when historically relevant. Selection was based on thematic relevance rather than formal criteria. **Conclusions**: Advancements in patient selection and pre-procedural planning, driven by enhanced imaging modalities such as CT pulmonary angiography (CTPA) and contrast-enhanced ultrasound, have led to improved outcomes and reduced complications. While the benefits of embolisation are well-documented, ongoing research continues to explore the long-term outcomes, including post-embolisation pulmonary function, recurrence rates, and quality of life improvements.

## 1. Introduction

### 1.1. Definition and Clinical Significance

Pulmonary arteriovenous malformations (PAVMs) are rare vascular anomalies characterised by direct connections between pulmonary arteries and veins, leading to a pathological right-to-left shunt within the pulmonary circulation [1]. PAVMs can vary significantly in both size and complexity, and in some cases, patients may present with over 100 lesions—classified as diffuse PAVMs [2]. Pulmonary arteriovenous malformations are classified as simple when a single feeding artery connects to a single draining vein, whereas lesions involving multiple feeding arteries and/or draining veins are designated as complex PAVMs [2].

First identified by Churton in 1897, PAVMs are estimated to affect around 1 in 2600 people based on limited prevalence data cited in the British Thoracic Society’s Clinical Statement on PAVMs [1,3]. Retrospective studies also suggest that PAVMs occur roughly twice as frequently in females as in males [1].

### 1.2. Etiology

Pulmonary arteriovenous malformations (PAVMs) arise from abnormalities in angiogenesis and vessel remodeling [1]. Normally, a primitive capillary plexus forms between arteries and veins and remodels into a mature vascular network [4]. In PAVMs, this process is disrupted, creating direct arteriovenous connections that bypass the pulmonary capillary system, causing right-to-left shunts [1].

A major cause of PAVMs is hereditary haemorrhagic telangiectasia (HHT), an autosomal dominant disorder affecting mucocutaneous surfaces, lungs, liver, gastrointestinal tract, and CNS, with a prevalence of around 1 in 10,000 [1,5,6,7,8]. Other risk factors include hepatopulmonary syndrome (HPS), where liver dysfunction leads to hypoxaemia via vasodilators like nitric oxide and angiogenic factors such as VEGF, promoting direct artery-to-vein connections [1,9]. Congenital heart defects, particularly in univentricular patients post-Glenn or Fontan procedures, also contribute by excluding hepatic venous blood from the pulmonary circulation, leading to abnormal pulmonary vascular remodeling and PAVM formation [10].

### 1.3. Historical Perspective of Embolisation as a Treatment

Historically, PAVMs were treated with surgical resection via thoracotomy and lobectomy [11,12]. While effective, this approach was invasive and carried high morbidity due to the removal of healthy lung tissue. It has largely been replaced by percutaneous transcatheter embolisation, a minimally invasive technique using coils to occlude abnormal vessels [12]. This method offers lower risk, faster recovery, and a clinical success rate of around 75%, making it the preferred first-line treatment [11]. This review highlights advancements in embolisation techniques and their role in improving patient outcomes.

## 2. Pathophysiology and Clinical Presentation of PAVMs

### 2.1. Right-to-Left Shunting and Hypoxaemia

PAVMs facilitate aberrant right-to-left shunting, wherein deoxygenated blood from the pulmonary arterial circulation bypasses the alveolar capillary interface and enters the systemic circulation without undergoing gas exchange [3]. This physiological derangement leads to a reduction in arterial oxygen level (PaO_2_) and oxygen saturation (SaO_2_), thereby resulting in hypoxaemia [3]. The severity of hypoxaemia is closely related to the extent of right-to-left shunting, with larger shunt volumes leading to greater impairment in oxygenation [3]. Typically, when 50% of the cardiac output is shunted, arterial oxygen saturation (SaO_2_) falls to approximately 60%; in comparison, a 30% shunt is associated with a saturation of around 80%, while a 10% shunt corresponds to roughly 90% saturation [3].

Interestingly, despite the significant reduction in oxygen saturation, patients with small PAVMs often do not experience noticeable dyspnoea, even during physical exertion or air travel [3]. This is largely attributed to various compensatory physiological mechanisms. Instead, they may present with non-specific symptoms such as dizziness, fatigue, or palpitations [3].

### 2.2. Loss of Capillary Filter Function and Paradoxical Emboli

Under normal physiological conditions, the pulmonary capillary network serves as a crucial biological filter, effectively removing microorganisms, microthrombi, and other particulate matter from the venous circulation [13]. In individuals with PAVMs, this filtering mechanism is bypassed due to direct arteriovenous connections, thereby increasing the risk of unfiltered embolic material entering the systemic circulation [14].This scenario predisposes patients to potentially serious complications, including cerebral abscesses and paradoxical emboli, which most often affect the cerebral circulation but can occur in any artery, potentially causing life- or limb-threatening events such as stroke or myocardial infarction [14]. Evidence shows that as many as 18% of patients with PAVMs suffer from stroke, and modelling data suggest that at least 25% of those with untreated PAVMs will experience a stroke before the age of 65 [15].

### 2.3. Cardiac Compensation and Heart Failure

The presence of PAVMs can also contribute to a hyperdynamic circulatory state characterised by increased cardiac output [16]. This occurs as the heart compensates for the right-to-left shunt and resultant hypoxaemia by increasing stroke volume and heart rate to maintain adequate tissue oxygen delivery [16]. In patients with extensive AVMs, this chronic volume overload can lead to high-output heart failure [16]. Cho et al. described cases of HHT patients developing high-output cardiac failure due to significant hepatic AVMs, but pulmonary AVMs can similarly impose a sustained burden on cardiac function [17]. Over time, the heart’s compensatory mechanisms may become insufficient, resulting in myocardial decompensation and clinically evident heart failure [16]. Echocardiography in affected patients typically demonstrates compensatory left ventricular (LV) dilatation and LV hypertrophy with preserved or high systolic function, while high-output heart failure may present predominantly as diastolic dysfunction. Unlike congenital left-to-right shunts (which more commonly cause marked pulmonary vascular remodeling and pulmonary hypertension), isolated PAVMs usually do not produce significant pulmonary hypertension.

Although PAVMs are rare, they can cause serious complications such as ischaemic stroke, myocardial infarction, cerebral or systemic abscesses, and, rarely, massive haemoptysis or haemothorax [1]. Pregnancy in affected individuals also carries higher risks, including maternal mortality [3].

Early recognition and diagnosis are crucial. Common features include epistaxis—the most frequent symptom—and respiratory or cardiovascular manifestations [1,3]. Dyspnoea, reported in 13 to 56% of cases, is more common in patients with large AVMs or digital clubbing [1]. Other signs may include pleuritic chest pain, palpitations, cough, murmurs, cyanosis, and reduced exercise tolerance [1]. Prompt identification, particularly in at-risk patients, is essential for timely evaluation and to reduce the risk of severe complications, making a high index of suspicion key.

### 2.4. Diagnostic Pathways

In terms of diagnosis, transthoracic contrast echocardiography (TTCE) and chest CT angiography are the primary modalities for screening and evaluating PAVMs in patients with HHT [18,19]. TTCE is the preferred screening tool, with a sensitivity of up to 98.6% [18]. However, chest CT angiography is considered the gold standard for diagnosis, offering superior anatomical resolution, accurate localisation (including multiple or diffuse lesions), and detailed characterisation of PAVMs (e.g., simple vs. complex) [19]. It also plays a crucial role in guiding embolisation planning and post-procedural follow-up [19]. In contrast, other tests, such as pulse oximetry, chest radiography, arterial PaO_2_, and shunt measurements, have significantly lower sensitivity and are not recommended as primary screening tools [18].

## 3. Conventional Embolisation Techniques

### 3.1. Indications and Patient Selection

The British Thoracic Society Clinical Statement on PAVM has clearly stated that all patients with PAVMs that are visible on imaging should be referred to interventional specialists skilled in embolisation procedures [3]. The criteria for embolisation of asymptomatic PAVMs have been a subject of ongoing debate, particularly following the introduction of the so-called “3 mm guideline,” which historically led some institutions to adopt a 3 mm size threshold as the minimum criterion for intervention [3].

In 1992, Rosenblatt and colleagues performed an investigative analysis on a cohort of 17 patients, each presenting with a singular dominant PAVM [20]. Their study identified that eight of these individuals demonstrated radiological evidence of cerebral infarctions on magnetic resonance imaging (MRI) scans, indicative of prior ischemic events [20]. Notably, four patients experienced clinically apparent strokes, with the feeding arteries supplying their PAVMs measuring within a range of approximately 2.9 to 4.5 mm in diameter [20]. These clinical and imaging findings contributed to the formulation of a practical threshold—commonly referred to as the “3 mm guideline”—which advocates for the consideration of therapeutic embolisation in PAVMs exhibiting feeding arteries equal to or exceeding this diameter [20]. Over time, this criterion has heavily influenced the clinical decision-making protocols related to the treatment of PAVMs, reinforcing its role as a pivotal reference point in vascular interventional strategies. Nonetheless, these size-based recommendations were reconsidered and ultimately withdrawn in 2006, as emerging evidence suggested that embolisation of smaller PAVMs could also confer clinical benefits [20]. Treating PAVMs with feeding arteries smaller than 3 mm offers the advantage of reducing the risk of complications, such as cerebral abscess from bacterial embolisation and stroke from paradoxical bland embolisation, as highlighted by Pollak et al. (2006), in addition to the consideration that smaller PAVMs might progress to further enlargement [21].

### 3.2. Overview of Traditional Materials

Embolisation is a minimally invasive therapeutic procedure to stop the arterial blood supply to the PAVM. The choice of embolic agent has long been a critical consideration due to safety concerns and complications associated with traditional materials, alongside the continuous development of new agents.

Coil embolisation has been regarded as the earliest and mainstay treatment for PAVM introduced in the 1990s [22,23]. A plethora of coil ingredients have been introduced, such as stainless steel, platinum, or Inconel (a nickel-based superalloy), which may be either bare or covered with fibers to enhance their ability to promote clot formation (thrombogenicity), with thrombogenesis representing their primary mechanism to achieve occlusion [23]. Fibered coils, in particular, have shown superior efficacy in promoting thrombus formation and have been associated with a trend towards lower recanalisation rates compared to bare coils [24]. However, due to variability in follow-up durations and sample sizes across studies, the differences in recanalisation rates have not reached statistical significance [24].

Recanalisation remains a concern with coil embolisation, with contributing factors including proximal coil placement, coil oversizing, and an insufficient number of coils used [25,26]. These limitations have highlighted the importance of careful device selection and technique in determining procedural success. In response to these challenges, the Amplatzer Vascular Plug (AVP) (St. Jude Medical, Minneapolis, MN, USA) was developed as an alternative embolic device, offering improved control, stability, and occlusion efficiency [27].

Designed to improve efficiency, a single AVP can often replace multiple coils, reducing procedure time, radiation exposure, and overall costs. The AVPs are a series of self-expanding vascular occlusion devices made from nitinol mesh [27]. They are another non-absorbable embolising agent as alternative to coils and were derived from the septal occlusion plugs initially designed for congenital heart defects by Dr Kurt Anton Amplatz [27,28]. AVP comes in 4 categories, known as AVP I–AVP IV, of which differ in size, shape, delivery strategies, and thrombogenicity [28].

Each AVP model consists of two main parts: the vascular plug itself and a delivery wire [28]. The plugs are constructed from braided nitinol, a material known for its self-expanding properties [27]. Radiopaque platinum marker bands are incorporated at both ends of the plug to enhance visibility under fluoroscopic guidance, facilitating accurate placement during the procedure [27]. These markers also improve the ease and clarity of device visualization during follow-up imaging, such as CT angiography. One of these bands includes a stainless-steel screw that connects the plug to the delivery cable. AVPs are typically upsized to be 30% to 50% larger than the diameter of the target vessel at the site of occlusion [27].

### 3.3. Embolisation Procedure Technique

During percutaneous intervention, vascular access is typically obtained via the right femoral vein under ultrasound guidance using a microintroducer set.

A vascular sheath facilitates wire advancement into the inferior vena cava, after which an angled pigtail catheter is positioned in the main pulmonary artery before selecting the appropriate pulmonary branch [29]. Contrast is injected via the pigtail catheter to map PAVMs, identify feeding arteries, and assess pulmonary vasculature, with pressure measurements performed as needed [29].

Catheter selection depends on the embolisation device: coil embolisation uses a selective catheter over a hydrophilic guidewire, while AVP deployment uses a guiding catheter over a stiff wire, with angiography confirming PAVM location and vessel anatomy [21,30]. Coil embolisation requires a coaxial microcatheter system for distal delivery, whereas AVPs are preloaded on a delivery cable and inserted directly through the guiding catheter [31,32].

AVPs are deployed under fluoroscopy and can be repositioned before final release, while coil deployment varies by type: pushable coils are inexpensive but non-repositionable, and injectable coils allow dense packing but carry a higher risk of non-target embolisation [29,33,34].

Pushable coils are deployed using the anchor or scaffold technique [34]. In the anchor method, the initial coil is secured in a side branch before proximal packing, while the scaffold technique uses oversized high radial force coils to form a framework, with softer coils layered inside; balloon occlusion may assist in large or high-flow vessels [35].

To conclude the procedure, a post-embolisation arteriogram is performed to confirm successful vessel occlusion, ensuring that the draining sac is no longer visible. Once adequate embolisation is confirmed, the catheter and sheath are withdrawn over a guidewire, and hemostasis is achieved through manual compression.

### 3.4. Short-Term and Long-Term Success Rate

Following successful embolisation and catheter removal, clinical outcomes further highlight the advantages of different embolic agents. In a review conducted by Wang et al., a total of 20 published studies described 340 feeding vessels treated with AVPs, reporting a recanalisation rate of approximately 1% [27]. This is notably lower than the recanalisation rates associated with coil embolisation, which range from 8% to 15% depending on the follow-up time period [27]. In addition to lower recanalisation rates, numerous studies have also demonstrated reduced PAVM persistence and reperfusion with AVPs compared to coil embolisation, further supporting the superiority of AVP embolisation [27].

Further supporting these findings, a study by Bortsford et al. evaluated 312 PAVM embolisations in 109 patients using non-fibered coils (NFCs), fibered coils (FCs), and AVPs [36]. All procedures were technically successful with no major complications. At 10-year follow-up, PAVM persistence-free survival was significantly higher in the AVP group (81.0%) compared to the 0.018-inch coil group (47.3%) (*p* < 0.0001), with no significant difference between NFCs and FCs [36]. Additionally, the AVP group had a much lower re-embolisation rate per PAVM (0.08) compared to coils (0.43) (*p* < 0.001), further emphasising the long-term effectiveness and durability of AVPs in PAVM treatment [36].

## 4. Innovations in Embolisation Materials

### 4.1. Newer Coil Design

Recent advancements in embolisation materials for pulmonary arteriovenous malformations (PAVMs) aim to improve occlusion durability, reduce recanalisation, and enhance procedural safety. Hydrogel-coated coils, with a platinum core and polymer that expands fourfold after around 20 min, enable dense, durable vessel occlusion and are MRI-compatible, facilitating follow-up and planning [37]. A prospective study by Iguchi et al. found them safe and effective [38]. They found that no recanalisation or reperfusion was observed during follow-up (100% occlusion durability), and only one serious adverse event (coil migration, grade 4) occurred, which was managed without sequelae, demonstrating high procedural safety.

Advances in delivery systems, particularly detachable coils, have improved procedural precision and safety. Available in larger diameters (up to 22 mm), they suit a wider range of anatomies [39]. Though more costly and potentially harder to deliver due to microcatheter friction, their controllability may improve outcomes [34]. Shimohira et al. reported higher persistent occlusion rates with high-volume, non-fibred detachable coils versus conventional pushable coils, supporting their long-term efficacy in PAVM embolisation [40].

**Figure 1:**
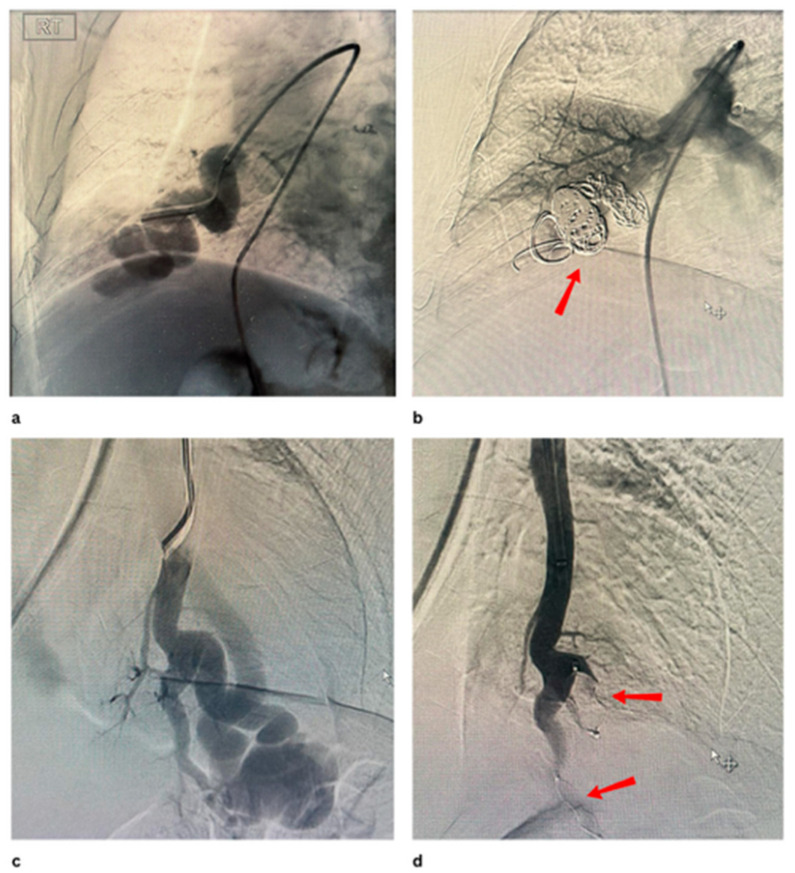
(**a**,**b**) show pre and post embolisation of the PAVM with multiple coils at the right lower lobe, (**c**,**d**) show pre and post embolisation ofthe complex PAVM with 2 AVPs at the left lower lobe.

**Figure 2:**
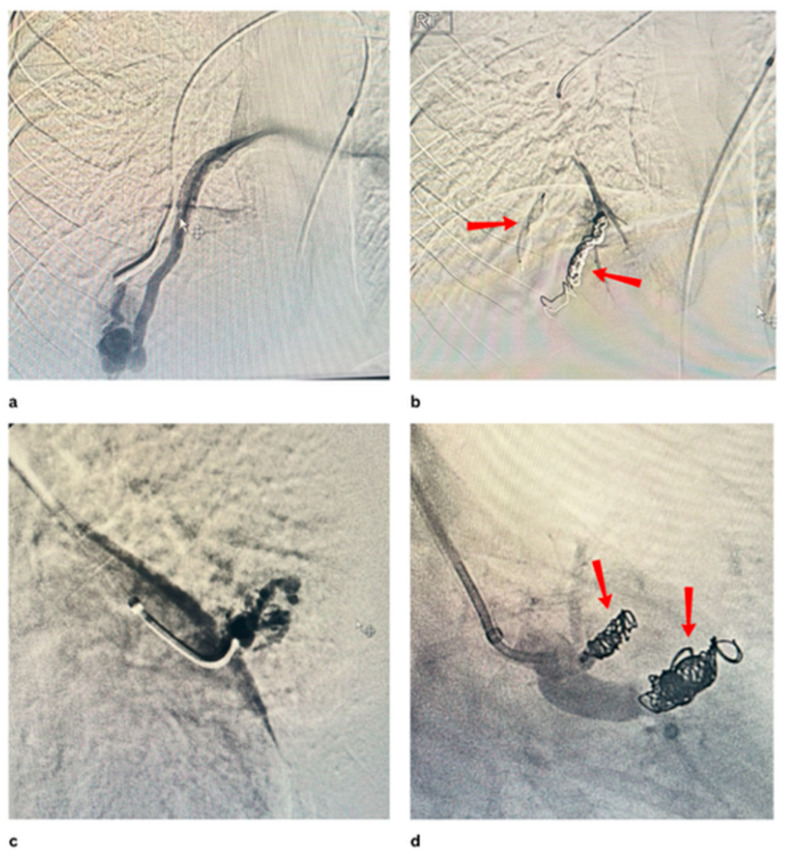
(**a**,**b**) show pre and post embolisation ofthe complex PAVM with an AVP and 2 coils at the right lower lobe. (**c**,**d**) show pre and post embolisation of the complex PAVM with 3 coils at the left side.

Figure 1 and Figure 2 show PAVMs embolised using detachable coils and/or AVP in simple and complex PAVMs, respectively.

### 4.2. Microvascular Plugs

Despite the advantages offered by coils—particularly hydrogel-coated and detachable variants—they may be suboptimal in cases involving high-flow shunts or larger-calibre vessels, where plug-based devices offer more effective occlusion. While the aforementioned AVP remains a widely used option in such scenarios, the Microvascular Plug (MVP) has emerged as a more versatile and navigable alternative for smaller or more tortuous anatomies.

Micro Vascular Plugs (MVPs) were introduced to clinical practice in 2013 [41]. MVP is a device specifically engineered for occluding small to medium-sized blood vessels [42]. It features an ovoid, self-expanding nitinol framework covered with polytetrafluoroethylene (PTFE) and is mounted on a push wire via a detachable screw mechanism. The device is available in four different sizes [43].

The fact that the MVP is coated with PTFE allows it to achieve immediate vessel occlusion even in patients undergoing procedural anticoagulation [44]. This coating, combined with the device’s design, also enables repositioning within the target vessel up to three times before final deployment, providing greater precision during placement [43]. The MVP offers several advantages in the treatment of PAVMs, including its compatibility with microcatheter delivery and the ability to be resheathed if necessary [43]. Furthermore, compared to coils, the MVP produces fewer metal artifacts on follow-up computed tomography (CT) scans, facilitating better post-procedural imaging assessment [44]. Importantly, the PTFE membrane may act as a barrier to prevent delayed recanalisation, a known issue after embolization with devices such as the AVPs and coils, potentially leading to improved long-term occlusion outcomes [44].

Multiple studies and early clinical experience have affirmed the effectiveness and safety of using the MVPs in the embolisation of PAVMs [44,45,46,47]. One of the most significant safety advantages is the MVP’s capacity for controlled deployment; its resheathable and repositionable nature significantly reduces the risk of non-target embolisation, which is a key concern when navigating complex pulmonary vasculature. Additionally, the MVP’s single-device deployment contrasts with coil embolisation, which often requires multiple devices, thereby reducing procedural time and radiation exposure [48]. Complication rates reported in MVP-based embolisations remain low, with minimal instances of device migration or post-procedural pulmonary infarction [48]. Moreover, long-term data on recanalisation rates are still evolving, although preliminary results are promising [44]. Overall, the MVP offers a robust option for PAVM management. However, much of the current evidence supporting these new devices is based on small single-centre or observational studies, limiting generalisability. Robust randomised or direct comparative studies are still lacking, so definitive superiority over existing coil embolisations cannot yet be confirmed. Larger multi-centre trials with longer follow-up are required to validate these promising results.

**Figure 3:**
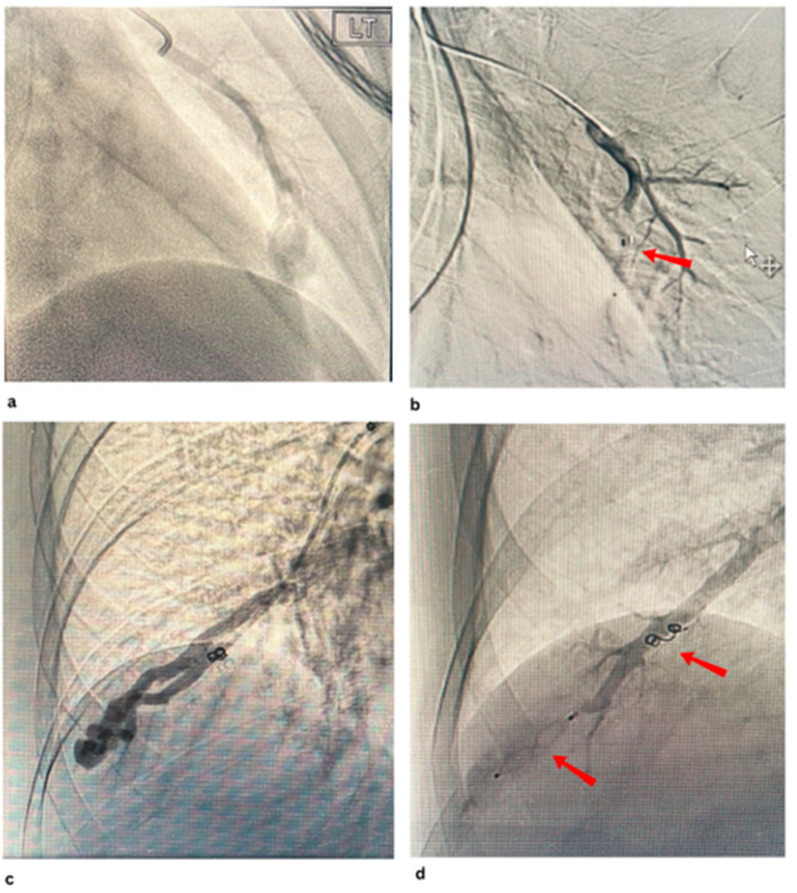
(**a**,**b**) show pre and post embolisation ofthe PAVM with an MVP at the left lingular lobe, (**c**,**d**) show pre and post embolisation of the complex PAVM with 1 AVP and a coil at the right lower lobe.

Figure 3a,b show embolisation of PAVM with MVP with occlusion of the feeding artery and further filling of the venous sac.

## 5. Imaging Advances Supporting Embolisation

### 5.1. High-Resolution CTA and 3D Reconstruction

Currently, high-resolution computed tomography angiography (CTA) has emerged as a highly effective, non-invasive alternative to traditional pulmonary angiography in the diagnosis of PAVMs [49]. It provides detailed visualisation of key anatomical components, including feeding arteries, draining veins, and the aneurysmal sac or nidus [49]. When combined with three-dimensional (3D) reconstruction, CTA significantly enhances pre-procedural planning by enabling virtual simulations of different interventional approaches [50]. This capability is particularly valuable in cases with complex or atypical congenital vascular anatomy, as it aids in selecting the most suitable strategy for coil embolisation. By optimising device selection and access routes, 3D reconstruction reduces procedural time and radiation dose while improving technical success rates, directly contributing to better patient outcomes.

### 5.2. Cone Beam CT for Intra-Procedural Guidance

Although high-resolution CTA with 3D reconstruction aids comprehensive preoperative mapping, real-time intraoperative visualisation remains significant. Cone beam CT (CBCT) has emerged as an invaluable tool in PAVM embolisation [51]. In fact, CBCT has been increasingly utilised across a wide range of endovascular procedures, extending beyond PAVM embolisation to include applications in renal artery and prostatic artery embolisation, not to mention the management of neurovascular conditions [52,53]. Real-time imaging with CBCT provides continuous visual guidance throughout the procedure—from accessing target vessels to catheter navigation and stent deployment—enabling faster decision-making and improved workflow efficiency [51]. This real-time guidance reduces the risk of non-target embolisation and procedural complications, increasing procedural safety and reducing the likelihood of reintervention.

### 5.3. Role of 4D Flow MRI in Assessing Haemodynamics Pre- and Post-Embolisation

Although CBCT excels at guiding interventions, complementary imaging modalities like 4D flow MRI are essential to assess the functional haemodynamic consequences of PAVM embolisation. Four-dimensional flow MRI is an advanced imaging technique that provides thorough time-resolved flow measurements across the entire field of view, independent of operator skill [54]. Unlike conventional MRI, it enables post hoc analysis of flow at any location and offers 3D visualization of important haemodynamic parameters such as wall shear stress (WSS, the tangential force of blood on vessel walls), oscillatory shear index (OSI, the degree of directional change in WSS), and energy loss (EL, the energy dissipated due to friction and turbulence) [54]. Specifically, in PAVM management, it allows dynamic assessment of blood flow patterns and shunt quantification before and after embolisation, facilitating evaluation of both disease severity and treatment success [54].

### 5.4. AI-Assisted Image Analysis and Segmentation

Artificial intelligence (AI), especially deep learning, can be employed to automate image analysis in PAVM embolisation. For example, convolutional neural networks have been effective in vascular segmentation and anomaly detection [55]. On top of that, a retrospective batch analysis conducted by Languis-Wiffen et al. has shown that AI algorithm has achieved a higher diagnostic accuracy for the detection of PE on CTPA in comparison with the attending radiologist [56]. This could be extrapolated to the case of PAVM embolisation where AI can effectively detect multiple PAVMs—especially the diffuse types that are challenging to pick up manually. Additionally, AI can accurately segment pulmonary vessels from CTA or MRI scans, swiftly and consistently distinguishing feeding arteries, draining veins, and the nidus, outperforming conventional manual methods [57]. By improving detection accuracy and reducing missed lesions, AI-assisted imaging may reduce clinical delays and prevent complications such as stroke or brain abscess, ultimately improving survival and quality of life.

## 6. Technique Modifications and Adjuncts

These imaging innovations not only enhance procedural planning but also lay the foundation for more refined and technically advanced embolisation strategies. As image-guided interventions continue to evolve, so too have the techniques and adjuncts used during PAVM embolisation.

### 6.1. Embolisation of Feeding Artery vs. Nidus Targeting

Targeting the feeding arteries has traditionally been the standard approach in PAVM embolotherapy, aiming to occlude the arterial inflow, thereby inducing thrombosis and collapse of the malformation [58]. While generally effective, this technique can present limitations, particularly in complex or diffuse PAVMs where multiple feeding arteries are involved [58]. In such cases, the procedure may become more time-consuming and technically challenging, increasing the risk of incomplete occlusion and recurrence [58].

To address these limitations, alternative strategies such as nidus targeting have been explored. A retrospective study by Hayashi et al., involving 21 patients, demonstrated a higher rate of reperfusion in lesions treated by feeding artery embolisation alone compared to those managed with nidus-targeted techniques [59]. This suggests that nidus-directed embolisation—particularly when combined with conventional techniques—may offer greater durability and reduce the likelihood of recurrence [60].

### 6.2. Dual Catheters Technique

Building on the need for more comprehensive and effective embolisation strategies, especially in complex or high-flow PAVMs, the dual catheter technique has emerged. Commonly used in neurovascular interventions, it has been adapted for PAVM embolisation to enhance control and safety [61]. This method involves the use of two microcatheters through a single access site, allowing for simultaneous delivery of embolic agents such as coils or liquid materials [62,63]. One catheter typically deploys an initial framing coil to anchor the position and prevent migration, while the second is used to densely pack additional coils or maintain distal access [62]. The initial coil remains undetached until a stable coil mass is achieved, minimising the risk of device displacement and ensuring a more durable occlusion [62]. This technique is particularly advantageous in high-flow lesions or wide-necked fistulas, where single-catheter approaches may not provide sufficient stability or packing density [64]. While a dual-microcatheter technique has been described for PAVM embolisation to position and stabilise coils, currently there are no large-scale data on its effect on recurrence or complications. Evidence from neurovascular procedures suggests technical success and safety, but these results may not fully apply to pulmonary vasculature, highlighting the need for dedicated PAVM studies. Despite its potential advantages, the dual-catheter approach may require additional training, longer procedural times, and careful cost consideration, particularly when implemented outside high-volume specialised centres.

### 6.3. Balloon-Occlusion Embolisation

Balloon-occlusion embolisation is an adjunctive strategy to enhance precision and safety in high-flow or complex PAVMs [20]. It involves temporary balloon inflation within the feeding artery to control blood flow, reducing the risk of paradoxical embolisation and coil migration [20]. In large or high-flow PAVMs, balloon occlusion improves procedural success by allowing controlled placement and dense packing of embolic materials, particularly detachable coils [65]. The initial coil can be held in place while additional coils are packed proximally, and gradual balloon deflation confirms positioning before final release, resulting in effective, durable occlusion [65,66]. Mori et al. demonstrated that this approach is safe and reliable for PAVM embolisation [67]. Chu et al. has also reported microballoon-occluded transcatheter embolisation in 38 PAVM patients, with no continued perfusion on follow-up CT and no non-target embolisation complications, further supporting the efficacy and safety of balloon-assisted techniques [68].

### 6.4. Navigation Tools (Robotic or Augmented Reality Platforms)

Beyond technical embolisation advances, recent developments focus on improving operator precision and spatial orientation through navigation tools like robotic-assisted systems and augmented reality (AR) platforms.

The CorPath GRX system, used in neurovascular and peripheral interventions, enables remote control of microcatheters and guidewires with fine micromovements, enhancing navigation through tortuous vessels and reducing operator fatigue [69]. Next-generation technologies include untethered active micro- and nanoscale robots that operate wirelessly [69]. Examples such as flow-driven magnetic swarms and magnetic coils can reach small, deep vessels beyond traditional catheter access, offering high manoeuvrability and precise embolic delivery, with potential to transform embolisation techniques [69].

## 7. Clinical Outcomes and Complications

With advances improving embolisation precision, assessing clinical outcomes and complications remains crucial. Success is evaluated both technically and clinically: immediate technical success is the complete absence of flow through the PAVM on angiography without additional embolic material, while follow-up success is determined by imaging—≥70% reduction in aneurysm or draining vein size on CT or persistent absence of flow on pulmonary angiography [70]. Clinically, improvements in oxygen saturation, symptoms, and exercise tolerance further confirm effectiveness [70].

Standard follow-up includes contrast-enhanced CT and transthoracic echocardiography at 3–6 months, with long-term surveillance tailored to conditions such as hereditary haemorrhagic telangiectasia (HHT) [1]. If residual flow is detected at 1-year follow-up, repeat CT pulmonary angiography, and additional embolisation may be needed [1]. Common complications include pleuritic chest pain (15–31%), especially with feeding arteries > 8 mm, usually self-limiting and managed with NSAIDs [71]. Other potential issues include coil migration, non-target embolisation, and rarely pulmonary hypertension, paradoxical embolism, haemoptysis, or lung infarction/infection [72,73].

## 8. Special Populations and Considerations

As embolisation advancement continues to progress, it is increasingly vital to tailor management strategies for specific populations and clinical contexts, such as pregnant patients and those with HHT.

### 8.1. Pregnancy

Pregnancy poses specific challenges in managing PAVMs due to physiological changes that can worsen right-to-left shunting and heighten the risk of serious complications such as PAVM rupture, haemothorax, and hypovolemic shock [74]. Therefore, the timing of embolisation is crucial for pregnant patients. Whenever feasible, elective embolisation is advised before pregnancy to minimize these risks [75]. Updated guidelines recommend treating symptomatic PAVMs during pregnancy, as the benefits of embolisation generally outweigh procedural risks. If a PAVM is detected during pregnancy, treatment must balance procedural and radiation risks against the dangers of leaving it untreated, with embolisation generally safest in the second and third trimesters [76].

### 8.2. Management in HHT

Patients with HHT require careful monitoring, as PAVMs are often asymptomatic until complications arise [77]. Symptomatic lesions, particularly those causing hypoxemia, should be treated to improve clinical outcomes, while small or asymptomatic lesions in children may be safely observed with close follow-up [77]. Lifelong surveillance is essential, typically with CT imaging 3 to 12 months after embolisation and then every 5 years to detect new, growing, or reperfused PAVMs [77]. Patient education, genetic counselling, including family screening, and early intervention are critical to optimise long-term outcomes [77].

## 9. Future Directions and Research Gaps

As the field of PAVM embolisation continues to evolve, attention is shifting towards innovative technologies and evidence-based refinements that can optimise outcomes and reduce recurrence. Advancements in modelling, materials, and data collection are paving the way for more personalised, predictive, and durable treatment strategies.

### 9.1. Role of Computational Fluid Dynamics and Personalised Modelling

Computational fluid dynamics (CFD) and personalised modelling are emerging tools to improve PAVM embolisation. CFD uses numerical algorithms to simulate fluid behaviour and is well established in cardiovascular and cerebrovascular research, but its application to PAVMs is still limited [78,79].

Personalised CFD models combine patient-specific CT anatomy with haemodynamic data from 4D flow MRI to simulate blood flow [80]. For coils, CFD can model how increased packing density raises nidus resistance, reducing flow velocity and pressure—a factor linked to lower recanalisation in cerebral aneurysms and potentially beneficial in PAVMs [81]. CFD visualisation of haemodynamic changes can aid treatment planning and may reduce complications or recurrences [81,82]. Despite promise, its use in PAVMs is exploratory, limited by imaging resolution, challenges in reconstructing nidus geometry, and lack of standardized quantitative tools, highlighting key areas for future research [81,82].

### 9.2. Predictive Modelling for Recanalisation Risk

Recanalisation remains a major challenge after PAVM embolisation, potentially causing symptom recurrence, paradoxical emboli, or neurological complications. Current follow-up often lacks risk stratification, highlighting the need for predictive models to guide surveillance and personalise management. Studies in related vascular domains, such as intracranial aneurysms and AVMs, have explored models using factors like aneurysm size, rupture status, first coil packing density (FCP), and angiographic outcomes [83]. Models by Ogilvy et al. and He et al. improved the prediction of aneurysm recurrence using multivariate or machine learning approaches, which may apply to PAVMs [83,84,85]. In AVM embolisation, factors like access technique, ethanol dilution, AVM type, and glue usage were linked to complication risk, supporting integration of procedural and anatomical variables into risk models. While predictive tools for PAVMs remain limited, adapting these frameworks could help identify high-risk patients and guide embolisation strategies [86]. Future directions include multicentre datasets, validating machine learning algorithms, and improved nidus characterisation through advanced imaging.

## 10. Drug-Eluting Embolic Materials

Building on advancements in embolisation, recent research has explored innovations beyond mechanical occlusion, notably drug-eluting embolic materials.

These agents combine mechanical occlusion with localized drug delivery and have been primarily studied in oncology [87]. They illustrate dual therapeutic potential: obstructing abnormal vasculature while modulating the local tissue environment [87,88]. In PAVMs, drug-eluting embolics could theoretically minimise post-embolisation inflammation, promote vessel fibrosis, or deliver antiangiogenic agents to reduce recanalisation risk. However, this drug-eluting embolic materials concept for treating PAVM remains largely unexplored, with no clinical trials or case series assessing their safety, efficacy, or long-term outcomes in PAVM treatment. Challenges such as device availability, regulatory approval, and cost must be addressed before clinical adoption can be considered.

### Long-Term Registry and Future Research

In summary, current evidence continues to support the efficacy and safety of PAVM embolization. According to the meta-analysis by Shahin et al. (2025), simple PAVM morphology, younger age, and use of plugs were independently associated with higher long-term treatment success, while coil use and combined feeding-artery/sac embolisation predicted lower durability [89]. They also found no statistically significant difference in recanalisation rates between different embolisation materials, underscoring that long-term registries are crucial to detect rare adverse events or device-specific complications [89]. Building on these emerging technologies and treatment concepts, long-term registries and real-world outcome data are essential to validate safety, efficacy, and durability in PAVM embolisation [89,90,91]. These registries capture large-scale patient outcomes, identifying patterns in recurrence and complications over time. Alongside ongoing and future clinical trials of novel embolic agents and predictive models, this data will drive evidence-based, personalised management strategies for PAVM patients.

## References

[1] Danyalian A., Hernandez F. (2021). Pulmonary Arteriovenous Malformation. StatPearls.

[2] Yuranga Weerakkody D’Souza D. Pulmonary Arteriovenous Malformation. Radiopaedia.org. 2 May 2008. https://radiopaedia.org/articles/pulmonary-arteriovenous-malformation?lang=gb.

[3] Shovlin C.L., Condliffe R., Donaldson J.W., Kiely D.G., Wort S.J. (2017). British Thoracic Society Clinical Statement on Pulmonary Arteriovenous Malformations. Thorax.

[4] Udan R.S., Culver J.C., Dickinson M.E. (2013). Understanding vascular development. Wiley Interdiscip. Rev. Dev. Biol..

[5] Locke T., Gollamudi J., Chen P. (2022). Hereditary Hemorrhagic Telangiectasia (HHT). StatPearls.

[6] Cullivan S., Kevane B., McCullagh B., O’Connor T.M., Condliffe R., Gaine S. (2024). Pulmonary vascular manifestations of hereditary haemorrhagic telangiectasia. Pulm. Circ..

[7] Donaldson J.W., McKeever T.M., Hall I.P., Hubbard R.B., Fogarty A.W. (2014). The UK prevalence of hereditary haemorrhagic telangiectasia and its association with sex, socioeconomic status and region of residence: A population-based study. Thorax.

[8] McDonald J., Pyeritz R.E. (2017). Hereditary Hemorrhagic Telangiectasia. Adam MP.

[9] Bansal K., Gore M., Mittal S. (2021). Hepatopulmonary Syndrome. StatPearls.

[10] Kavarana M.N., Jones J.A., Stroud R.E., Bradley S.M., Ikonomidis J.S., Mukherjee R. (2014). Pulmonary arteriovenous malformations after the superior cavopulmonary shunt: Mechanisms and clinical implications. Expert Rev. Cardiovasc. Ther..

[11] Bakhos C.T., Wang S.C., Rosen J.M. (2016). Contemporary role of minimally invasive thoracic surgery in the management of pulmonary arteriovenous malformations: Report of two cases and review of the literature. J. Thorac. Dis..

[12] Meek M.E., Meek J.C., Beheshti M.V. (2011). Management of Pulmonary Arteriovenous Malformations. Semin. Interv. Radiol..

[13] Yuan Y. (2019). Vascularized lung tissue engineering. Encyclopedia of Tissue Engineering and Regenerative Medicine.

[14] Si-Mohamed S.A., Cierco A., Gamondes D., Restier L.M., Delagrange L., Cottin V., Dupuis-Girod S., Revel D. (2022). Embolization of Recurrent Pulmonary Arteriovenous Malformations by Ethylene Vinyl Alcohol Copolymer (Onyx^®^) in Hereditary Hemorrhagic Telangiectasia: Safety and Efficacy. J. Pers. Med..

[15] Shovlin C.L. (2014). Pulmonary Arteriovenous Malformations. Am. J. Respir. Crit. Care Med..

[16] Liao Y., Chen K.H., Huang G.Y., Song W. (2014). Pulmonary arteriovenous malformations presenting as refractory heart failure. J. Thorac. Dis..

[17] Cho D., Kim S., Kim M., Seo Y.H., Kim W., Kang S.H., Park S.-M., Shim W. (2012). Two Cases of High Output Heart Failure Caused by Hereditary Hemorrhagic Telangiectasia. Korean Circ. J..

[18] Saboo S.S., Chamarthy M., Bhalla S., Park H., Sutphin P., Kay F., Battaile J., Kalva S.P. (2018). Pulmonary arteriovenous malformations: Diagnosis. Cardiovasc. Diagn. Ther..

[19] Lacombe P., Lacout A., Marcy P.Y., Binsse S., Sellier J., Bensalah M., Chinet T., Bourgault-Villada I., Blivet S., Roume J. (2013). Diagnosis and treatment of pulmonary arteriovenous malformations in hereditary hemorrhagic telangiectasia: An overview. Diagn. Interv. Imaging.

[20] Shimohira M., Kawai T., Ohta K. (2023). An Update on Embolization for Pulmonary Arteriovenous Malformations. Interv. Radiol..

[21] Pollak J.S., Saluja S., Thabet A., Henderson K.J., Denbow N., White R.I. (2006). Clinical and Anatomic Outcomes after Embolotherapy of Pulmonary Arteriovenous Malformations. J. Vasc. Interv. Radiol..

[22] Hartnell G.G., Jackson J.E., Allison D.J. (1990). Coil embolization of pulmonary arteriovenous malformations. Cardiovasc. Interv. Radiol..

[23] Baba Y. (2023). Embolisation coils and microcoils. Radiopaediaorg.

[24] Liebig T., Henkes H., Fischer S., Weber W., Miloslavski E., Mariushi W., Brew S., Kühne D. (2004). Fibered Electrolytically Detachable Platinum Coils Used for the Endovascular Treatment of Intracranial Aneurysms: Initial Experiences and Mid-Term Results in 474 Aneurysms. Interv. Neuroradiol..

[25] Hong J., Lee S.Y., Cha J.G., Lim J.K., Park J., Lee J., Cha S.-I., Kim C.-H., Seo H. (2021). Pulmonary arteriovenous malformation (PAVM) embolization: Prediction of angiographically-confirmed recanalization according to PAVM Diameter changes on CT. CVIR Endovasc..

[26] Sue M.J., Luong T.T., Park J., Ding P.-X., Hao F., Eghbalieh N., Lee E.W. (2022). A Multicenter, Retrospective, Matched, Comparison Study of Clinical Efficacy and Cost-Effectiveness of Caterpillar Arterial Embolization Device versus Fibered Coils in Arterial Embolization. Appl. Sci..

[27] Wang W., Li H., Tam M.K., Zhou D.Y., Wang D., Spain J. (2012). The Amplatzer Vascular Plug: A Review of the Device and its Clinical Applications. Cardiovasc. Interv. Radiol..

[28] Loffroy R., Chevallier O., Mazit A., Malakhia A., Midulla M. (2023). AmplatzerTM Vascular Plugs for Embolisation: A 10-Year Single-Centre Retrospective Study. J. Clin. Med..

[29] (2022). How I Do It: Pulmonary Arteriovenous Malformations: A Summary of Interventional Management. Endovascular Today. https://evtoday.com/articles/2022-apr/how-i-do-it-pulmonary-arteriovenous-malformations-a-summary-of-interventional-management.

[30] Prasad S.N., Sharma S., Singh V., Phadke R.V. (2022). Endovascular management of pulmonary arteriovenous malformations presenting as multiple brain abscesses. BMJ Case Rep..

[31] Teitelbaum G.P., Reed R.A., Larsen D., Lee R.K., Pentecost M.J., Finck E.J., Katz M.D. (1993). Microcatheter Embolization of Non-neurologic Traumatic Vascular Lesions. J. Vasc. Interv. Radiol..

[32] Guneyli S., Cinar C., Bozkaya H., Parildar M., Oran I. (2014). Applications of the Amplatzer Vascular Plug to various vascular lesions. Diagn. Interv. Radiol..

[33] Oka S., Kohno S., Arizono S., Onishi Y., Fumimoto M., Yoshida A., Ishikura R., Ando K. (2024). Enhancing precision in vascular embolization: Evaluating the effectiveness of the intentional early detachment technique with detachable coils in complex cases. CVIR Endovasc..

[34] Xiao N., Lewandowski R.J. (2022). Embolic Agents: Coils. Semin. Interv. Radiol..

[35] (2025). OpenAthens. Sign in Oclc.org. https://www-sciencedirect-com.knowledge.idm.oclc.org/science/article/pii/S1089251608000085.

[36] Botsford A., Tradi F., Loubet A., Tantawi S., Soulez G., Giroux M.-F., Faughnan M.E., Gauthier A., Perreault P., Bouchard L. (2024). Transarterial Embolization of Simple Pulmonary Arteriovenous Malformations: Long-Term Outcomes of 0.018-Inch Coils versus Vascular Plugs. J. Vasc. Interv. Radiol..

[37] Ferral H. (2015). Hydrogel-Coated Coils: Product Description and Clinical Applications. Semin. Interv. Radiol..

[38] Iguchi T., Hiraki T., Matsui Y., Fujiwara H., Sakurai J., Baba K., Toyooka S., Gobara H., Kanazawa S. (2020). Embolization using hydrogel-coated coils for pulmonary arteriovenous malformations. Diagn. Interv. Imaging.

[39] (2025). Embolization of a Large Pulmonary Arteriovenous Malformation. Endovascular Today. https://evtoday.com/articles/2014-apr-supplement/embolization-of-a-large-pulmonary-arteriovenous-malformation.

[40] Mathevosian S., Sparks H., Cusumano L., Roberts D., Majumdar S., McWilliams J. (2024). Embolization of De Novo Pulmonary Arteriovenous Malformations Using High-Volume Detachable Non-Fibered Coils: Propensity-Matched Comparison to Traditional Coils. J. Clin. Med..

[41] (2013). BIBA Publishing. MVP Microvascular Plug for Peripheral Embolization Gets the CE Mark. Interventional News. https://interventionalnews.com/mvp-microvascular-plug-for-peripheral-embolization-gets-the-ce-mark/.

[42] (2015). The MVPTM Micro Vascular Plug: A New Paradigm in Peripheral Embolization. Endovascular Today. https://evtoday.com/articles/2015-apr/the-mvp-micro-vascular-plug-a-new-paradigm-in-peripheral-embolization.

[43] Mailli R., Chevallier O., Mazit A., Malakhia A., Falvo N., Loffroy R. (2023). Embolisation Using Microvascular Plugs for Peripheral Applications: Technical Results and Mid-Term Outcomes. Biomedicines.

[44] Conrad M.B., Ishaque B.M., Surman A.M., Kerlan RKJr Hope M.D., Dickey M.A., Hetts S.W., Wilson M.W. (2015). Intraprocedural safety and technical success of the MVP Micro Vascular Plug for embolization of pulmonary arteriovenous malformations. J. Vasc. Interv. Radiol..

[45] Ratnani R., Sutphin P.D., Koshti V., Park H., Chamarthy M., Battaile J., Kalva S.P. (2019). Retrospective comparison of pulmonary arteriovenous malformation embolization with the polytetrafluoroethylene-covered nitinol microvascular plug, AMPLATZER plug, and coils in patients with hereditary hemorrhagic telangiectasia. J. Vasc. Interv. Radiol..

[46] Latif M.A., Bailey C.R., Motaghi M., Areda M.A., Galiatsatos P., Mitchell S.E., Weiss C.R. (2023). Postembolization Persistence of Pulmonary Arteriovenous Malformations: A Retrospective Comparison of Coils and Amplatzer and Micro Vascular Plugs Using Propensity Score Weighting. Am. J. Roentgenol..

[47] Shahin Y., Gill A., Lejawka A., Willis R., Vijayakumar C., Abbas M., Kusumawidjaja D. (2024). Embolization Outcomes of Pulmonary Arteriovenous Malformations: A 10-Year Experience from a Tertiary Referral Center. Arab. J. Interv. Radiol..

[48] Giurazza F., Ierardi A.M., Contegiacomo A., Corvino F., Carrafiello G., Niola R. (2021). Embolization with MVP (Micro Vascular Plug^®^): Experience on 104 patients in emergent and elective scenarios. CVIR Endovasc..

[49] Shin S.M., Kim H.K., Crotty E.J., Hammill A.M., Wusik K., Kim D.H. (2020). CT Angiography Findings of Pulmonary Arteriovenous Malformations in Children and Young Adults With Hereditary Hemorrhagic Telangiectasia. Am. J. Roentgenol..

[50] Kato Y., Sano H., Katada K., Ogura Y., Hayakawa M., Kanaoka N., Kanno T. (1999). Application of three-dimensional CT angiography (3D-CTA) to cerebral aneurysms. Surg. Neurol..

[51] Barral M., Chevallier O., Cornelis F.H. (2023). Perspectives of Cone-Beam Computed Tomography in Interventional Radiology: Techniques for Planning, Guidance, and Monitoring. Tech. Vasc. Interv. Radiol..

[52] Park S.J., Cho Y., Lee H.N., Lee S., Chung H.H., Park C.H. (2024). Enhancing procedural decision making with cone beam CT in renal artery embolization. Sci. Rep..

[53] Raz E., Nossek E., Sahlein D.H., Sharashidze V., Narayan V., Ali A., Esparza R., Peschillo S., Chung C., Diana F. (2023). Principles, techniques and applications of high resolution cone beam CT angiography in the neuroangio suite. J. Neurointerv. Surg..

[54] Hyodo R., Takehara Y., Mizuno T., Ichikawa K., Horiguchi R., Kawakatsu S., Ebata T., Naganawa S., Jin N., Ichiba Y. (2023). Four-dimensional Flow MRI Assessment of Portal Hemodynamics and Hepatic Regeneration after Portal Vein Embolization. Radiology.

[55] Yamashita R., Nishio M., Do R.K.G., Togashi K. (2018). Convolutional neural networks: An overview and application in radiology. Insights Imaging.

[56] Langius-Wiffen E., de Jong P.A., Hoesein F.A.M., Dekker L., van den Hoven A.F., Nijholt I.M., Boomsma M.F., Veldhuis W.B. (2023). Retrospective batch analysis to evaluate the diagnostic accuracy of a clinically deployed AI algorithm for the detection of acute pulmonary embolism on CTPA. Insights Imaging.

[57] Mank Q.J., Thabit A., Maat A.P.W.M., Siregar S., van Walsum T., Kluin J., Sadeghi A.H. (2025). Artificial intelligence-based pulmonary vessel segmentation: An opportunity for automated three-dimensional planning of lung segmentectomy. Interdiscip. Cardiovasc. Thorac. Surg..

[58] Müller-Hülsbeck S., Marques L., Maleux G., Osuga K., Pelage J.P., Wohlgemuth W.A., Andersen P.E. (2020). CIRSE Standards of Practice on Diagnosis and Treatment of Pulmonary Arteriovenous Malformations. Cardiovasc. Interv. Radiol..

[59] Hayashi S., Baba Y., Senokuchi T., Nakajo M. (2012). Efficacy of Venous Sac Embolization for Pulmonary Arteriovenous Malformations: Comparison with Feeding Artery Embolization. J. Vasc. Interv. Radiol..

[60] Srinivas S., Roberts D.G., McWilliams J.P., Cusumano L.R. (2025). Feeding-Artery Microvascular Plug Embolization Versus Nidus-Plus-Feeding-Artery Coil Embolization of Pulmonary Arteriovenous Malformations. J. Clin. Med..

[61] Hirano Y., Koizumi S., Shojima M., Ishikawa O., Kiyofuji S., Umekawa M., Saito N. (2023). Double-catheter technique for the embolization of recurrent cerebral aneurysms: A single-center experience. Surg. Neurol. Int..

[62] Greben C.R., Setton A., Putterman D., Caplin D., Lenner R., Gandras E.J. (2013). Pulmonary Arteriovenous Malformation Embolization: How We Do It. Tech. Vasc. Interv. Radiol..

[63] Abecassis I.J., Nerva J.D., Ghodke B.V., Sekhar L.N., Levitt M.R., Kim L.J. (2017). The dual microcatheter technique for transvenous embolization of dural arteriovenous fistulae. J. Neurointerv. Surg..

[64] Baxter B., Rosso D., Lownie S. (1998). Double microcatheter technique for detachable coil treatment of large, wide-necked intracranial aneurysms. Am. J. Neuroradiol..

[65] Kanematsu M., Kondo H., Goshima S., Tsuge Y., Watanabe H., Moriyama N. (2012). Giant High-Flow Type Pulmonary Arteriovenous Malformation: Coil Embolization with Flow Control by Balloon Occlusion and an Anchored Detachable Coil. Korean J. Radiol..

[66] Cil B.E., Erdogan C., Akmangit I., Cekirge S., Balkanci F. (2004). Use of the TriSpan Coil to Facilitate the Transcatheter Occlusion of Pulmonary Arteriovenous Malformation. Cardiovasc. Interv. Radiol..

[67] Mori K., Shiigai M., Saida T., Anno I., Wada M., Minami M. (2008). A Modified Metallic Coil Embolization Technique for Pulmonary Arteriovenous Malformations Using Coil Anchors and Occlusion Balloon Catheters. Cardiovasc. Interv. Radiol..

[68] Chu H.H., Kim G.H., Gwon D.I. (2024). An Alternative Endovascular Technique for Treatment of Pulmonary Arteriovenous Malformation: Microballoon-occluded Transcatheter Embolization Using n-butyl-2-cyanoacrylate. Cardiovasc. Interv. Radiol..

[69] Mendes Pereira V., Rice H., De Villiers L., Sourour N., Clarencon F., Spears J., Tomasello A., Hernandez D., Cancelliere N.M., Liu X.Y.E. (2024). Evaluation of effectiveness and safety of the CorPath GRX robotic system in endovascular embolization procedures of cerebral aneurysms. J. Neurointerv. Surg..

[70] Peng Y., Liu X., Chan K.F., Song X., Zhang L. (2025). Robotic–Assisted Endovascular Embolization: Progress and Future Perspectives. SmartBot.

[71] Letourneau-Guillon L., Faughnan M.E., Soulez G., Giroux M.F., Oliva V.L., Boucher L.M., Dubois J., Prabhudesai V., Therasse E. (2010). Embolization of Pulmonary Arteriovenous Malformations with Amplatzer Vascular Plugs: Safety and Midterm Effectiveness. J. Vasc. Interv. Radiol..

[72] Chamarthy M.R., Park H., Sutphin P., Kumar G., Lamus D., Saboo S., Anderson M., Kalva S.P. (2018). Pulmonary arteriovenous malformations: Endovascular therapy. Cardiovasc. Diagn. Ther..

[73] Lanza E., Gennaro N., Poretti D., Straffi L., Marcheselli S., Tramarin M., Pedicini V. (2019). Full recovery after non-target cerebral embolization of n-butyl-cyanoacrylate occurred during emergency treatment of a facial arteriovenous malformation. CVIR Endovasc..

[74] Haitjerm T., ten Berg J.M., Overtoom T.T.C., Ernst J.M.P.G., Westermann C.J.J. (1996). Unusual Complications After Embolization of a Pulmonary Arteriovenous Malformation. Chest.

[75] Di Guardo F., Presti V.L., Costanzo G., Zambrotta E., Di Gregorio L.M., Basile A., Palumbo M. (2019). Pulmonary Arteriovenous Malformations (PAVMs) and Pregnancy: A Rare Case of Hemothorax and Review of the Literature. Case Rep. Obstet. Gynecol..

[76] (2025). Redirect Notice. Google.com. https://curehht.org/guidelines/pregnancy/.

[77] Majumdar S., McWilliams J.P. (2020). Approach to Pulmonary Arteriovenous Malformations: A Comprehensive Update. J. Clin. Med..

[78] Narsinh K.H., Ramaswamy R., Kinney T.B. (2013). Management of Pulmonary Arteriovenous Malformations in Hereditary Hemorrhagic Telangiectasia Patients. Semin. Interv. Radiol..

[79] (2012). Computational Fluid Dynamics. An overview|ScienceDirect Topics. Sciencedirect.com. https://www.sciencedirect.com/topics/materials-science/computational-fluid-dynamics.

[80] Reid L., Rea P.M. (2021). An Introduction to Biomedical Computational Fluid Dynamics. Advances in Experimental Medicine and Biology.

[81] Wang Q., Guo X., Stäb D., Jin N., Poon E.K.W., Lim R.P., Ooi A. (2022). Computational fluid dynamic simulations informed by CT and 4D flow MRI for post-surgery aortic dissection—A case study. Int. J. Heat Fluid Flow.

[82] Wiśniewski K., Tomasik B., Tyfa Z., Reorowicz P., Bobeff E.J., Stefańczyk L., Posmyk B.J., Jóźwik K., Jaskólski D.J. (2021). Porous Media Computational Fluid Dynamics and the Role of the First Coil in the Embolization of Ruptured Intracranial Aneurysms. J. Clin. Med..

[83] Zhang B., Chen X., Zhang X., Ding G., Ge L., Wang S. (2024). Computational modeling and simulation for endovascular embolization of cerebral arteriovenous malformations with liquid embolic agents. Acta Mech. Sin..

[84] Aoki K., Nagashima H., Murayama Y. (2025). Risk factors for recanalization after coil embolization for cerebral aneurysms: Importance of the first coil and prediction model. J. Stroke Cerebrovasc. Dis..

[85] Ogilvy C.S., Chua M.H., Fusco M.R., Reddy A.S., Thomas A.J. (2015). Stratification of Recanalization for Patients With Endovascular Treatment of Intracranial Aneurysms. Neurosurgery.

[86] He T., Chen K., Chen R.D. (2023). A predictive model for the recurrence of intracranial aneurysms following coil embolization. Front. Neurol..

[87] Park K.B., Do Y.S., Kim D.I., Kim Y.W., Park H.S., Shin S.W., Cho S.K., Hyun D.-H., Choo S.W. (2019). Endovascular treatment results and risk factors for complications of body and extremity arteriovenous malformations. J. Vasc. Surg..

[88] Mikhail A.S., Negussie A.H., Mauda-Havakuk M., Owen J.W., Pritchard W.F., Lewis A.L., Wood B.J. (2021). Drug-eluting embolic microspheres: State-of-the-art and emerging clinical applications. Expert Opin. Drug Deliv..

[89] Shahin Y., Vijayakumar C., Gill A., Lejawka A., Bennett S., Willis R., Abbas M., Kusumawidjaja D. (2025). A Meta-Analysis and Meta-Regression of Embolisation Outcomes of Pulmonary Arteriovenous Malformations. Cardiovasc. Interv. Radiol..

[90] Trotter J.P. (2002). Patient registries: A new gold standard for “real world” research. Ochsner J..

[91] Pisa F., Arias A., Bratton E., Salas M., Sultana J. (2023). Real world data for rare diseases research: The beginner’s guide to registries. Expert Opin. Orphan Drugs.

